# Automated Calibration of RSS Fingerprinting Based Systems Using a Mobile Robot and Machine Learning

**DOI:** 10.3390/s21186270

**Published:** 2021-09-18

**Authors:** Marcin Kolakowski

**Affiliations:** Institute of Radioelectronics and Multimedia Technology, Warsaw University of Technology, Nowowiejska 15/19, 00-665 Warsaw, Poland; m.kolakowski@ire.pw.edu.pl; Tel.: +48-22-234-7635

**Keywords:** positioning, SLAM, fingerprinting, BLE, machine learning, neural networks

## Abstract

This paper describes an automated method for the calibration of RSS-fingerprinting-based positioning systems. The method assumes using a robotic platform to gather fingerprints in the system environment and using them for training machine learning models. The obtained models are used for positioning purposes during the system operation. The presented calibration method covers all steps of the system calibration, from mapping the system environment using a GraphSLAM based algorithm to training models for radio map calibration. The study analyses four different models: fitting a log-distance path loss model, Gaussian Process Regression, Artificial Neural Network and Random Forest Regression. The proposed method was tested in a BLE-based indoor localisation system set up in a fully furnished apartment. The results have shown that the tested models allow for localisation with accuracy comparable to those reported in the literature. In the case of the Neural Network regression, the median error of robot positioning was 0.87 m. The median of trajectory error in a walking person localisation scenario was 0.4 m.

## 1. Introduction

The market for indoor location-based services is growing rapidly. We can now find many localisation systems applications ranging from professional solutions installed in industrial environments to more casual ones used in public spaces.

The indoor location services can be supplied using systems based on multiple technologies. The applications requiring high accuracy and precision are usually based on ultra-wideband (UWB) technology, which, under the right conditions, can achieve an accuracy of a few centimetres [1]. Unfortunately, the UWB technology is expensive in terms of the cost of the devices, which makes its use limited to professional solutions.

Most of the less accuracy-demanding location-based services can be fulfilled using one of the narrowband communications standards. The most popular standards used for positioning are Wi-Fi and Bluetooth Low Energy (BLE). The popularity of those standards allows for the development of widely accessible systems (especially smartphone-based), which can be used for multiple purposes ranging from indoor pedestrian navigation to personnel or patient positioning.

The localisation in narrowband systems is usually performed based on the Received Signal Strength (RSS) measurements. There are two prominent positioning methods used in those systems: RSS-ranging and fingerprinting.

In RSS-ranging, the measured power value is converted to distance between the localised tag and pieces of the system infrastructure based on one of the multiple propagation models [2]. The distances are then processed using one of the typical positioning algorithms such as trilateration [3], or Unscented Kalman Filter [4].

The second, more popular method is fingerprinting [5], in which the tag is localised through a comparison of the registered RSS values with the radio map containing information about signal power distribution in the system deployment area. The radio map is a database created at system set-up and composed of fingerprints including RSS measurement results and the location where they were taken.

The fingerprinting method has multiple advantages. First of all, its implementation is easy, as in its simplest form, it only requires the sharing of the radio map with the system users for comparison purposes. Additionally, as it does not require information on the location of the system infrastructure, it can easily be implemented in already existing systems and Wi-Fi networks (e.g., navigation systems at the airports or university campuses).

The most significant disadvantage of fingerprinting is the effort required to construct the radio map used for localisation. In a typical scenario, the radio map is created manually by taking multiple measurements in the area covered by the system. As the performance of the method is dependent on the map density [6] and a number of conducted measurements [7], the construction of an accurate radio map may be a lengthy and tiresome process, especially in large areas. Additionally, the radio maps often become outdated when even small changes are introduced into the system environment (e.g., moving an access point or placing additional pieces of furniture). Therefore, maintaining high localisation accuracy would require frequent calibration.

The time and effort needed for fingerprinting systems calibration cause several problems. First of all, they might significantly raise the system installation and maintenance costs, limiting those systems applications to more professional uses. Additionally, the need for lengthy calibration might prevent the use of fingerprinting-based systems in several specific applications requiring fast and temporary system installation at many locations, for example, medical trials where the patients’ activity is monitored at their homes.

The costs and effort related to the fingerprinting system calibration can be greatly reduced through the use of crowdsourcing algorithms [8]. The crowdsourcing methods assume using a vast user-collected set of RSS samples and are typically implemented in WiFi-smartphone–based localisation systems with many users. The results are typically gathered during the system routine operation and usually include the measured RSS values and additional sensor readings such as results from smartphones’ inertial sensors [9] or LiDAR scans in case of collecting the results using robots [10]. The gathered data are then processed to create a high-quality map of the area.

Another way to speed up system calibration is to measure the RSS values in a limited number of reference points and interpolate a complete radio map by fitting a propagation model or training machine learning algorithms [11]. Such an approach can also be used to improve localisation accuracy in systems, where the fingerprints are gathered in a traditional way [12].

This paper presents an automated method for indoor radio map calibration, which adopts both of the above approaches. The method uses a mobile platform to gather the RSS fingerprints in multiple locations in the area covered by the system. The gathered data are then used to estimate radio map based on a propagation model or to train a machine learning algorithm, which are then used for localisation.

The radio map calibration is performed in two steps. First, the system environment is mapped using the developed SLAM algorithm. The algorithm is based on the GraphSLAM framework and was developed with small, cluttered living spaces in mind. The second step is gathering the fingerprints by driving the robot through the apartment. The obtained RSS values and their LiDAR-estimated locations are processed to estimate a radio map and create models for localisation using four different methods: fitting the log-distance path loss model parameters and training a Gaussian Process, Neural Network and Random Forest regressors.

The method’s efficiency was assessed in a Bluetooth Low Energy based positioning system deployed in a fully furnished apartment. The results have shown that the method can be successfully used in indoor localisation scenarios. As the method was tested in a BLE-based system, for the remainder of the paper, “RSS” means “BLE RSS” if not stated otherwise.

The paper makes the following contributions:

1. A description of a complete method of RSS-based fingerprinting positioning system calibration is presented. The method goes through all steps, from environment mapping to radio map estimation;
2. Four different, popular methods are tested for system calibration. The study analyses the performance of exponential path-loss model fitting and standard machine learning methods: Gaussian Process Regression, Neural Networks and Random Forest Regression;
3. The concept of radio map calibration is tested in a typical living environment (a furnished apartment), which is not common [13]. In most works, the experiments are performed in office spaces, where the nature of the received power changes is different than in smaller, cluttered environments. Therefore, the results may be valuable to the teams working on the positioning solutions intended for individual use [14], for example, Ambient and Assisted Living systems monitoring the daily activity of people with dementia;
4. The experimental data (LiDAR scans, RSS measurements for several scenarios) gathered during the described study are posted in an online repository [15].

## 2. Related Works

The problem of reducing the costs of fingerprinting systems calibration attracts the attention of multiple research groups. As the majority of systems implemented in public spaces, where crowdsourcing data is relatively easy, use Wi-Fi technology, most relevant works concern such systems. Works investigating Bluetooth-based systems are much less popular. However, as both systems rely on power levels measurements, the presented methods are usually easily transferable between the technologies.

Processing crowdsourced datasets is a demanding task, which requires solving two problems:

- estimation of the crowdsourced fingerprints locations;
- interpolation of a radio map or training a model for localisation.

In the most common crowdsourcing methods intended for use in smartphone-based systems, the user location is usually derived based on the smartphone sensors reading, for example, from inertial units [16] or magnetometers [9]. In [16], the user is localised using a dead-reckoning algorithm with pulling the positioning results to characteristic points of the environment like doorways or narrow corridors. A similar approach is used in [6,17], where the characteristic landmarks used to improve the accuracy are building entrances, doorways and corridor intersections. The passing through such a point is detected based on compass measurements and estimation of the traveled distance.

A more advanced localisation scheme is presented in [9], where the location of the fingerprints is derived with a dead-reckoning based GraphSLAM. The novelty of the proposed algorithm is a loop-closure achieved using magnetic field measurements and observed Wi-Fi signals similarity. The GraphSLAM framework is also used in [18]. Aside from using inertial-based constraints, the proposed solution introduces a concept of so-called “Virtual Wi-Fi landmarks” located at the typical trajectory turns. The estimated distance from the virtual landmarks is used as an additional constraint in graph optimisation.

Some of the smartphone-based solutions use only the registered RSS values. In [19], the fingerprint locations are estimated with a radio map currently used by the system and additionally filtered. The solution requires calibration of a coarse radio map ahead of using the system. A much more advanced solution is analysed in [20], where the radio map is constructed based on unlabelled signatures set using Multi-Dimensional Scaling enabled by estimating pairwise distances between the data points using trilateration.

Fingerprint locations can also be determined using other concurrently working localisation systems. In [21], a separate UWB-based positioning system is used. The research presented in [22] uses a hybrid BLE/UWB system.

The localisation of the fingerprints is easier to infer in scenarios in which the data is gathered using mobile robots. An example of a robot’s use in a BLE based fingerprinting system is presented in [23], where a line-following LEGO robot was used to gather fingerprints. More advanced solutions were tested in Wi-Fi systems. In [10], the robot builds a map of the area, segments it and plans a path, which it later traverses, measuring RSS values. Similar solutions are presented in [24,25], where the robots localise themselves based on the LiDAR (Light Detection and Ranging) data.

The second part of the crowdsourcing problem is estimating the radio map or training an appropriate model for localisation. The easiest way is to directly use the gathered fingerprints as the radio map as in [17,21,25,26]. Another simple solution is to average the fingerprints gathered in the same or very close locations [6,16].

The data can also be used to fit the parameters of the radio propagation models [22]. The paper [24] presents an adaptive signal model fingerprinting (ASMF) algorithm, in which the parameters of path loss, fading and shadowing models along with the access points (AP) locations are estimated and used to interpolate the radio map.

A similar solution is presented in [13], where the Radial Basis Function is used to estimate the BLE power distribution in an apartment. The method assumes performing multiple updates to the radio map by gathering additional fingerprints in areas where localisation accuracy is low.

More advanced interpolating signal power distribution use machine learning (ML) methods, of which Gaussian Process Regression (GPR) seems to be most prevalent in crowdsourcing applications. An example of the regressor’s use is presented in [10], where the data gathered by the robot is used to fit a GPR model estimating the power distribution in the system area. The GPR is also used in [9] to create an environment map integrating three measurement types: Wi-Fi signal levels, magnetic field strength and light intensity. A variation of GP is presented in [19], where the marginalised particle extended Gaussian Process (MPEGP) is used to filter noisy labels and update the radio map.

Another example of keeping the radio map up to date is described in [27], where Gradient Boosted Decision Tree regression is used to detect access points alterations and update the fingerprint database.

The machine learning methods can also be directly used for positioning purposes. There are several papers investigating the use of ML instead of a traditional radio map. The ML can localise the objects by solving classification or regression problems.

The first approach treats the localisation process as a classification problem, in which the location of the device must be assigned to a particular class being either a singular point [11,28] or a larger area such as a room [29]. The proposed methods use different types of classifiers: Support Vector Machine (SVM) [11], Random Forest [11,21,28] and Artificial Neural Networks (including Convolutional ones) [11,12,29].

The latter approach is to use the ML to solve a regression problem, where the results are the coordinates of the localised device. The studies described in the literature typically utilise Neural Networks [21,30,31,32] and k-Nearest Neighbours (kNN) [11,33].

A comparison of the positioning errors reported in the literature is presented in Table 1. The table omits some of the referenced works, as the classification-based methods assess the performance as the correct class assignment percentage, which is not directly comparable with the distance metrics.

The typical median localisation error of the RSS-based fingerprinting methods is in the range of 1–2 m. Better accuracy is reported only in a few of the analysed works. The reported results might not prove these methods superiority as the obtained results are impacted on many factors such as the test site, the number of reference anchors or a way of calibration data gathering.

In [13] (mean error of 0.6 m), the data for radio map interpolation is gathered manually in a specific way. After the initial localisation tests, the regions, where the localisation accuracy was the lowest are additionally sampled and the procedure repeated, until the accuracy is at an acceptable level. In the case of the UWB assisted solutions [22] (trajectory error in 0.42–0.52 m range), [21] (mean error in 0.72–0.85 m range), the number of gathered signatures is large and gathered by the person who later takes part in the tests. Additionally the tests in [21] were performed in a single room, which limited the negative impact of the through the wall propagation. The tests described in [23] (median error of 0.72 m) were performed in two places—single room and an office space. The presented results are a joint error CDF estimation for both of the locations.

In case of the Wi-Fi systems, sub-meter median errors were reported in cases, where either the number of the anchors was very high [19] (100 available access points—21 best chosen for localisation) or the test conditions were very specific [24] (large area partitioned into smaller rooms using low drywall walls).

From the above works, only a few were conducted in a living environment or a similar cluttered area [13,22,33], or using robots [10,23,24,25]. These methods will be the main yardstick for the proposed solution’s accuracy.

## 3. Method Description

**Figure 1:**
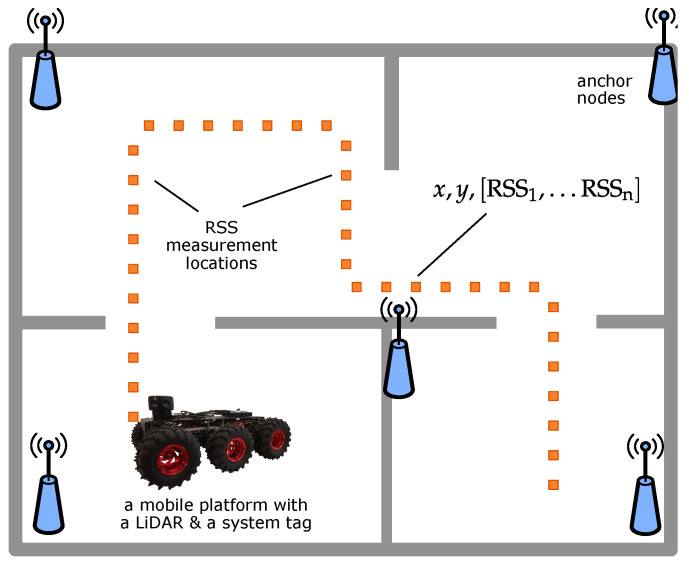
The concept of the proposed RSS system calibration method.

The concept of the proposed RSS positioning system calibration method is presented in Figure 1.

The proposed method assumes using a mobile robotic platform equipped with a LiDAR sensor and a system tag. In the proposed method, the system calibration is performed in two steps:

- environment mapping;
- RSS radio map calibration.

In the first step, the environment is mapped using the attached LiDAR. The robot is driven through the environment and takes stationary scans of the area. The gathered scans and odometry data are processed using the proposed SLAM algorithm. The resulting map is then used to localise the robot in the consecutive step and might be a basis for calibration path planning. The mapping step can be omitted if a map of the environment is already available.

The RSS radio map calibration consists of driving the platform through the system deployment area. At the same time, the robot localises itself using the LiDAR and concurrently measures the strengths of signals transmitted by the system infrastructure and received by the tag. The obtained results (RSS measurements) are saved along with the derived locations of the platform (x,y). The obtained database is then used to calibrate the localisation system and create a complete radio map or an ML model for future localisation.

## 4. System Environment Mapping

The first phase of the proposed RSS positioning system calibration method is mapping the environment using a mobile platform equipped with a LiDAR sensor. The basic steps of the proposed SLAM algorithm are presented in Algorithm 1.
**Algorithm 1** Environment mapping procedure **Input:** Scans $S=[s_{0}\cdots s_{N}]$, Odometry measurements $O=[o_{0}\cdots o_{N}]$ **Output:** Occupancy GridMap of the area *m*1:P← odometryConstraints(O)2:T←[]      ▹ initialise empty list for matching results3:**for***i* in range (0, N-1) **do**4:    t← ICP_matching($s_{i}$, $s_{i+1}$, $P_{i}$)        ▹ See Algorithm 25:    *T*.insert(*t*)6:X← graphSLAM(*S*, *T*, *P*)            ▹ See Algorithm 37:m← occupancyGridMap(*S*, *X*)

The procedure consists of several steps. First, the robot poses and geometric constraints between the consecutive scans are estimated based on the odometry readings. Next, the consecutive scans are matched with an Iterative Closest Point (ICP)-based algorithm using the obtained constraints as initial transformation estimation. Then, the odometry-based poses and computed transformations are used to create a graph and the robot poses are computed more accurately using a GraphSLAM-based algorithm. Finally, the scans and the poses are processed to build an occupancy grid map of the environment.

The input data of the algorithm are a set of scans registered in the system deployment area $S=[s_{1}\cdots s_{N}]$ and odometry measurement results, which allow estimating geometric constraints between the robot poses in which the scans were taken $O=[o_{1}\cdots o_{N}]$ (travelled distances *d* and heading changes Δθ). The algorithm assumes that the scans are taken when the robot is stationary, and a 360-degree LiDAR is used, but it can be easily adapted to other scenarios.

The first step of the algorithm is estimating robot poses and constraints between the consecutive scans based on odometry measurements. The pose is defined as a vector containing robot location and its heading direction:

(1) $$p=\left[\begin{array}{c} x\\ y\\ \theta \end{array}\right],$$

where *x*, *y* are robot coordinates and θ is an angle denoting its heading direction. Assuming the starting robot pose to be $p_{0}=\left[\begin{array}{ccc} 0 & 0 & 0 \end{array}\right]$, the poses and constraints between them can be estimated as follows:

(2) $$\begin{array}{c} p_{n}=\sum_{i=1}^{n}\left[\begin{array}{c} d_{i}\cos\left(\sum_{j=1}^{i}\Delta \theta_{i}\right)\\ d_{i}\sin\left(\sum_{j=1}^{i}\Delta \theta_{i}\right)\\ \Delta \theta_{i} \end{array}\right] \end{array}$$

(3) $$\begin{array}{c} t_{n,n-1}=\left[\begin{array}{ccc} d_{n} & 0 & \Delta \theta_{n} \end{array}\right], \end{array}$$

where $d_{i}$ and $\Delta \theta_{i}$ are traveled distance and heading change between poses *i* and i−1 and $t_{n,m}$ is an initial guess on transformation matching consecutive scans *n* and n−1.

### 4.1. Scan Matching

The goal of scan matching is to find a transformation $t_{m,n}$ which allows to align two scans *m*,*n* so that the common parts overlap. In the proposed algorithm the transformation is defined as a vector:

(4) $$t_{m,n}=\left[\begin{array}{ccc} t_{x} & t_{y} & \Delta \theta \end{array}\right],$$

where $t_{x}$, $t_{y}$ are translation in *x* and *y* axes and Δθ is the rotation angle between the scans. To combine the two scans, scan *n* is transformed to scans *m* coordinate system with:

(5) $$\begin{array}{c} s_{n}^{t}=H\left(t\right)s_{n}^{HC} \end{array}$$

(6) $$\begin{array}{c} H\left(t\right)=\left[\begin{array}{ccc} \cos\Delta \theta & -\sin\Delta \theta & t_{x}\\ \sin\Delta \theta & \cos\Delta \theta & t_{y}\\ 0 & 0 & 1 \end{array}\right], \end{array}$$

where $s_{n}^{t}$ is the transformed scan and $s_{n}^{HC}$ is a matrix containing the scan *n* points expressed in Homogenous Coordinates [34].

In the proposed method, the scans are matched using the Iterative Closest Point (ICP) algorithm [35], which finds the transformation minimising the distance between the points in the matched scans. In the proposed implementation, the matching is performed based on corresponding line segments of both scans. The complete procedure of scan matching is described in Algorithm 2.
**Algorithm 2** Scan matching procedure
 
**Input:** Scans $s_{m},s_{n}$, initial transformation guess *t*, number of ICP iterations *N*

 **Output:** transformation *t*
1:**for***i* in range (1, N) **do**2:    transformScan($s_{n}$, *t*)3:    $L_{m},L_{n}\leftarrow$ extract line features from $s_{m}$ and $s_{n}$4:    $P_{m},P_{n}\leftarrow$ find corresponding lines and points in $L_{m},L_{n}$ sets5:    t← ICP(*t*, CP)       ▹ match corresponding points using ICP6:**return***t*

**Figure 2:**
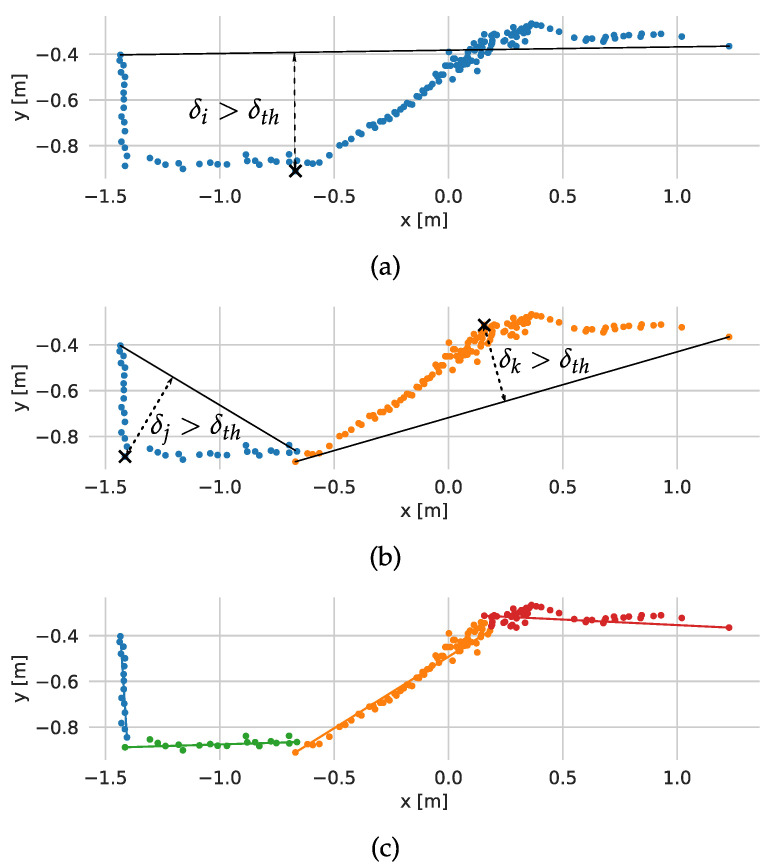
The steps of the split and merge method: (**a**) first splitting, (**b**) second splitting, (**c**) result of the algorithm.

The scan matching starts with transforming scan *n* based on the most recent transformation estimation *t* and extraction of lines from both scans. In the first iteration, the odometry estimate is used. The extraction is performed with a fast split-and-merge method, which is illustrated with Figure 2.

The split-and-merge method starts with dividing the scan into a few sets, processed separately, based on the LiDAR measurement angle. The method consists of estimating the line’s parameters connecting the first and the last point in the set and calculating the distance of the scan points from it. If the distance of the farthest point is larger than a defined threshold $\delta_{th}$ as in Figure 2a, the set is split at that point and the above procedure is repeated (Figure 2b). If all points are closer than $\delta_{th}$, it is assumed that they belong to one line and its parameters (slope *a*, y-intercept *b*) are estimated. The collinear segments are then merged. In order to avoid taking into account small segments detected in noisy point clouds, the method imposes additional requirements for the line: minimum length $l_{min}$ and minimum number of scan points $n_{min}$.

The next step is finding the corresponding lines and points in both scans. The lines are compared based on their range *r* and bearing ϕ with respect to the robot, which are derived from the line parameters:

(7) $$\begin{array}{c} r=\frac{\left|b\right|}{\sqrt{a^{2}+1}} \end{array}$$

(8) $$\begin{array}{c} \phi =atan2\left(\frac{-ab}{a^{2}+1},\frac{b}{a^{2}+1}\right). \end{array}$$

**Figure 3:**
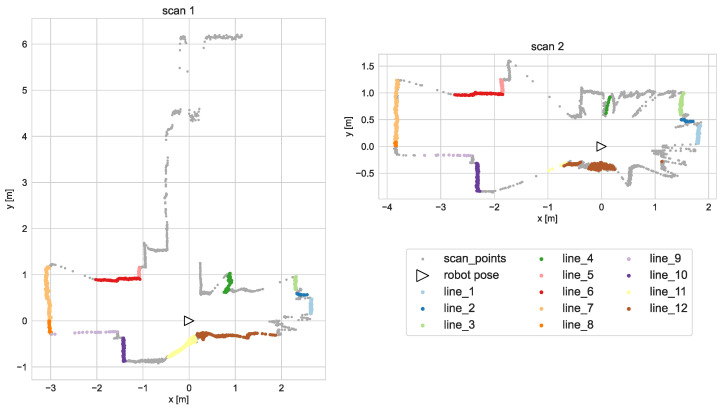
Exemplary scans with corresponding lines and points marked.

The lines *i*, *j* are treated as corresponding, when the following conditions are satisfied:

(9) $$\begin{array}{c} \left|r_{i}-r_{j}\right|<\Delta r_{th} \end{array}$$

(10) $$\begin{array}{c} \pi -\left|\left|\phi_{i}-\phi_{j}\right|-\pi \right|<\Delta \phi_{th}, \end{array}$$

where $\Delta r_{th}$ and $\Delta \phi_{th}$ are defined thresholds for range and bearing differences. Due to the platform moving, the scans might include different segments of the same line (e.g., different parts of the same wall). To avoid situations, when the ICP algorithm tries to match distant points, only the points, which minimum distance to the corresponding points set is smaller than a defined threshold Δp are matched. Exemplary scans with corresponding lines and points are presented in Figure 3.

Finally, the corresponding points are matched together using the ICP algorithm. The algorithm was implemented using a Levenberg–Marquardt–based Least Squares (LS) estimator. The LS minimises the following:

(11) $$\min_{t}\sum_{i=1}^{C}\frac{\sum_{j=1}^{N_{i}}min(||P_{m}^{i}-H\left(t\right)P_{n,j}^{i}\left||\right)}{N_{i}},$$

where *C* is the number of corresponding line pairs, $N_{i}$ is the number of points from line *i* of scan *n* chosen for fitting, $P_{m}^{i}$ is an array containing points from a corresponding line in scan *m*, H(t) is the transformation matrix and $P_{n,j}^{i}$ is a single point, for which the minimum distance is computed.

### 4.2. GraphSLAM

**Figure 4:**
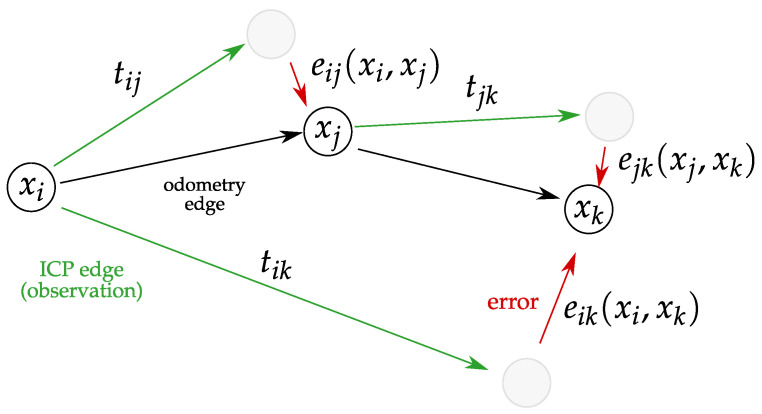
Exemplary graph used for robot pose optimisation.

The robot poses obtained using odometry and the results of consecutive scans fitting are used to construct a pose graph, which exemplary structure is presented in Figure 4.

The graph nodes are robot poses. The poses can be connected with two kinds of edges:

- odometry edges—constraints computed from odometry measurements;
- ICP edges—constraints obtained via ICP scan matching.

The GraphSLAM algorithm aims to optimise robot poses to reduce the errors between them and the poses resulting from the ICP observations. The main steps of the algorithm are presented in Algorithm 3.
**Algorithm 3** Scan matching procedure
 
**Input:** transformations *T*, odometry poses *P*, algorithm iterations *N*

 
**Output:** robot poses *X*
1:G← createGraph(*P*,*T*)2:**for***i* in range (1, N) **do**3:    C← getICPCandidates(G)         ▹ find scan pairs possible for matching4:    **for** *c* in C **do**5:        t← ICP_matching($s_{i}$, $s_{j}$, *G*)       ▹ match scans from each candidates pair6:        *G*.addICPEdge(t)7:    *G*.optimise()8:X←*G*.getRobotPoses()9:**return***X*

The algorithm starts with graph creation. Initially, the graph contains only the odometry and ICP edges between the consecutive scans. Aside from the graph structure, the algorithm creates occupancy grid maps for all of the poses and scans taken in them.

In the next step, the graph is analysed in order to determine which scan pairs can be efficiently matched. In the proposed implementation, the scans are considered good candidates for matching when:

- the poses are closer than $\Delta x_{th}$;
- the percentage of common area in the corresponding grid maps is higher than $c_{G}$.

The values of the above thresholds might be changed between the algorithm iterations to add new observations to the graph gradually.

The candidate scans are then matched using the ICP algorithm described in Section 4.1 and the obtained transformations are added to the graph as observation edges. When there are no more edges to add, the graph is optimised.

The goal of the graph optimisation is to reduce the errors between the robot poses estimated based on the observations and the ones stored in the graph. In the proposed implementation the poses are optimised with an LS-estimator minimising the global error vector, which is a concatenation of individual error vectors $e_{i}$ estimated for each observation edge:

(12) $$e_{i}=t2v\left(H{\left(T_{i}\right)}^{-1}H{\left(X_{m}^{\left(i\right)}\right)}^{-1}H\left(X_{n}^{\left(i\right)}\right)\right),$$

where t2v is a function converting a transformation matrix (6) to a vector, $T_{i}$ is a transformation associated with an observation *i* and $X_{m}^{\left(i\right)}$, $X_{n}^{\left(i\right)}$ are the poses, which are connected by the analysed observation edge.

After the optimisation is complete, the graph and accompanying grid maps are updated and the procedure is repeated for a given number of times or until there are no new observation edges to add.

The obtained poses are then used to construct the complete occupancy grid map of the system deployment area. The map is a reference for robot localisation. In the study a particle-filter–based localisation algorithm [36] was used.

## 5. RSS Radio Map Calibration

The second phase of the proposed method is RSS radio map calibration, which consists of two steps:

- data gathering;
- RSS model fitting.

In the first step, the calibration data are gathered by driving the robotic platform across the apartment collecting RSS measurement samples in multiple locations. The obtained dataset includes the measured RSS values alongside measurement locations determined by the robot based on the LiDAR scans, odometry, and the created map of the surroundings.

The dataset is used to train and fit models, which will be used during the typical system operation to localise objects and users using the fingerprinting method. The presented study analyses the use of the following models:

- log-distance path loss model;
- Gaussian Process regression;
- Neural Network;
- Random Forests Regression.

The first two models are used to estimate the system’s radio map. In such a case, the user localisation is computed using K-nearest neighbours, a typical fingerprinting algorithm. The two latter methods result in complete models, which take the measured RSS values as an input and return the user’s location.

All of the above models are frequently used in the literature. In the following study, their performance is evaluated for calibrating the system deployed in a small furnished apartment.

The collected calibration data are preprocessed before fitting the models. The collected signatures are binned based on the measurement location using a square grid of 0.1 m spacing. The fingerprints are then averaged, which reduces measurement noise and helps prevent situations in which the models would be fitted to a large number of records gathered in a small area.

### 5.1. Log-Distance Path Loss Model

The log-distance path loss model (LDPL) is arguably the most popular propagation model used in RSS-ranging-based positioning systems. In the model, the power of the received signal is modelled as:

(13) $$RSS=RSS_{0}-10\gamma log\frac{d}{d_{0}},$$

where $RSS_{0}$ is the signal strength received at the reference distance $d_{0}$, *d* is the distance between the tag and the anchor and γ is the path loss exponent.

In the proposed method, the path loss exponent γ is fitted separately for each anchor. The reference power $RSS_{0}$ is measured at the system deployment. The fitting is performed using the Least-Squares based optimiser. The disadvantage of the model is that it requires information on the anchors locations.

### 5.2. Gaussian Process Regression

Gaussian Process regression (GPR) is a rapidly popularity-gaining class of machine learning algorithms. The goal of Gaussian Process algorithms, instead of estimating parameters of a single function, is to fit a probability distribution over multiple functions to fit the given data. In the analysed case, a separate GPR model is fitted for each anchor and is used to estimate the RSS value at a location *x*:

(14) $$RSS_{n}\left(x\right)∼GP\left(m\left(x\right),k\left(x,x^{\prime }\right)\right),$$

where m(x) is a mean function denoting the RSS value in point *x* and $k(x,x^{\prime })$ is a covariance function (also called a kernel function), which defines the relationship between values modelled in points *x* and $x^{\prime }$. In the implemented model a modified Matérn kernel is used:

(15) $$k\left(x,x^{\prime }\right)=\frac{1}{\Gamma \left(\nu \right)2^{\nu -1}}{\left(\frac{\sqrt{2\nu }}{l}d\left(x,x^{\prime }\right)\right)}^{\nu }K_{\nu }\left(\frac{\sqrt{2\nu }}{l}d\left(x,x^{\prime }\right)\right)+\sigma^{2}I+c,$$

where $d(x,x^{\prime })$ is the Euclidean distance between the two locations, $K_{\nu }(\dot{)}$ and $\Gamma (\dot{)}$ are the Bessel and gamma functions, respectively. The tunable parameters of the model are the length-scale parameter *l*, ν parameter controlling the smoothness of the function, additive white noise variance $\sigma^{2}$ and a constant value *c*.

### 5.3. Neural Network

**Figure 5:**
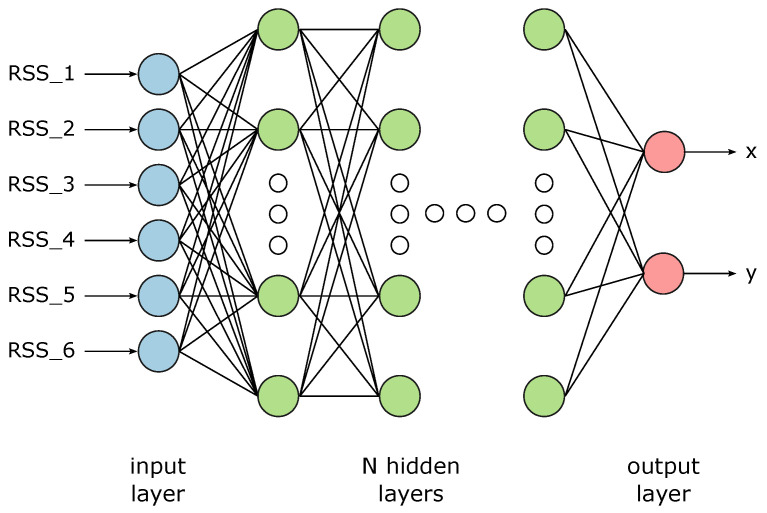
The architecture of the Artificial Neural Network used by the method.

Neural networks are arguably one of the most popular machine learning methods in use today. Deep learning finds multiple applications, from classifying and processing images to solving advanced regression problems. The proposed method uses a Feed-Forward Artificial Neural Network (ANN), which architecture is presented in Figure 5.

The input layer accepts RSS values measured by particular anchors. In the study’s case, the network has six inputs as the system infrastructure consists of six anchors. When using the method in other systems, the input dimension must be adjusted.

The network has multiple hidden layers, which number *N* is a tunable parameter. The number of neurons in the layers is also subject to optimisation. For both the input and hidden layers, the ReLu activation function is used.

The output of the ANN algorithm is the x-y coordinates of the localised device. Thus the output layer consists of two neurons with linear activation functions.

The network is trained based on the signatures gathered during the calibration phase. The training is performed in batches, for which size is optimised to achieve the best possible localisation accuracy.

### 5.4. Random Forest Regression

**Figure 6:**
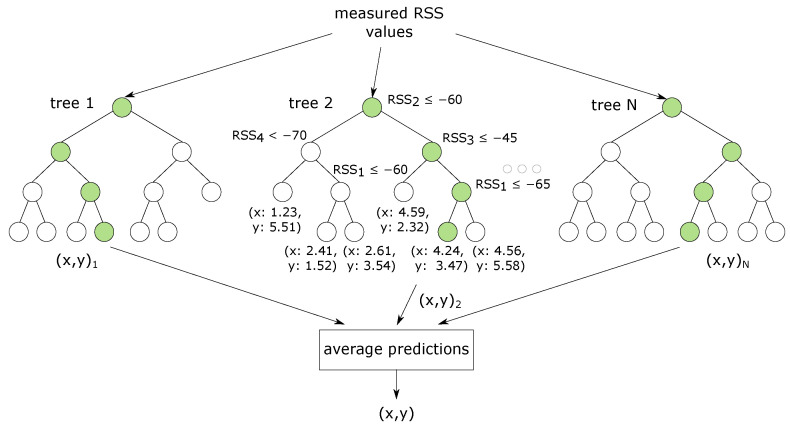
The architecture of the Random Forest Regressor used by the method.

Random Forest Regression (RFR) is an ensemble learning method, which combines predictions of multiple decision tree estimators. The architecture of the RFR model used in the study is presented in Figure 6

The result of the RFR is the average of the results returned by decision trees constituting the forest. The Decision Tree is a supervised machine learning method widely used to solve both classification and regression problems. The method consists of building the decision tree by recursively splitting the dataset based on data features (in this case based on RSS values measured by particular anchors) and thresholds determined based on the assumed strategy.

Typically, the dataset is initially split based on several features-threshold combinations and regression mean squared errors are evaluated for the resulting sets. Then a split is performed based on the combination, for which the error was the lowest. The procedure is performed only on sets, which size is larger than a defined threshold. Otherwise, the set is used to form a leaf representing the output of the decision tree.

The trees forming the random forest are formed based on the randomly chosen samples from the dataset. The three main tunable parameters of RFR are the number of decision trees in the forest *N*, the maximum number of features considered during the dataset split and the minimum data set split size.

## 6. Experiments

The proposed method was implemented in Python and tested with experiments using a BLE-based positioning system. The experiments consisted of the three following steps:

- mapping the system environment;
- radio calibration of the system;
- localisation of a moving robot and a walking person.

The measurement results gathered during the experiment can be found online in a Zenodo repository [15].

### 6.1. Measurement Location and Equipment

**Figure 7:**
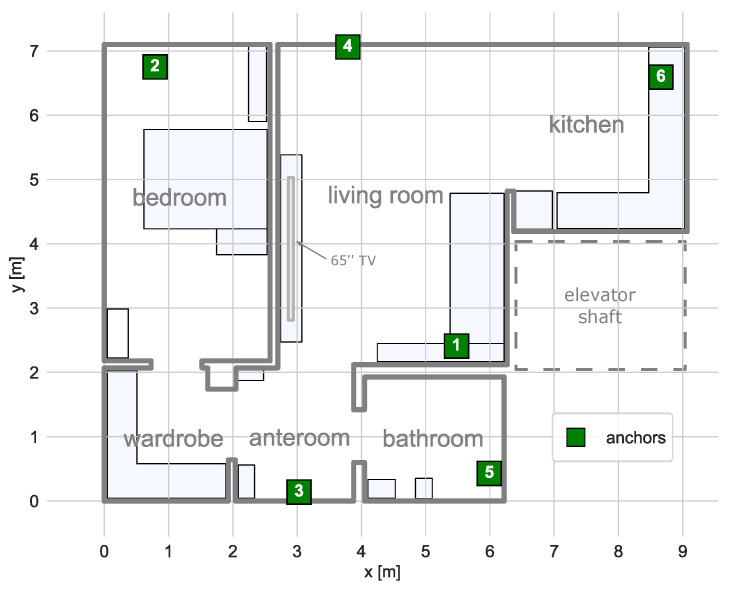
The plan of the experiment area and locations of the system anchors. The blue rectangles mark locations of the furniture pieces and appliances.

The experiment was conducted in a fully furnished apartment. The system used in the study is described in more detail in [4]. The plan of the apartment and locations of the system infrastructure is presented in Figure 7.

The apartment consisted of two rooms, a kitchen, a bathroom and a small wardrobe and an anteroom. The system infrastructure used in the study comprised six anchors. The anchors were equipped with two Laird BL652 modules with external antennas of perpendicular polarization. In the system, the signal transmission is reversed in comparison to the method description. The tag transmits BLE packets five times per second in three advertisement channels. The anchors periodically switch the reception channels and measure the power of the received signals. The results from both modules are averaged and sent to a localisation server, which stores them in a database for future processing.

**Figure 8:**
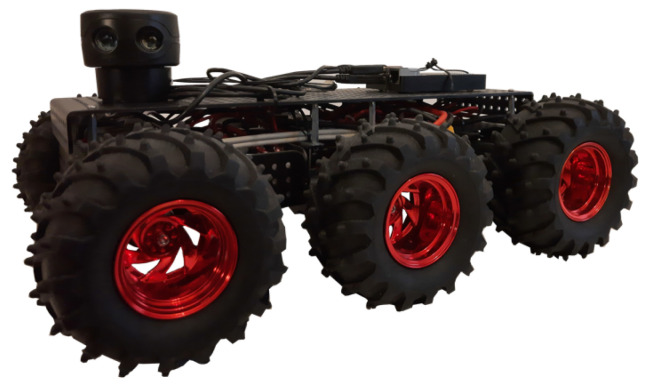
The mobile platform used in the study.

The robotic platform used in the study is presented in Figure 8.

The platform was based on the Dagu Wild Thumper 6WD chassis and was controlled with a specially developed Python-based controller running on Raspberry Pi 4. The platform’s main advantage is its size and a high maximum payload of 5 kg; thus, it can be equipped with multiple additional devices. Additionally, its high suspension allows the platform to pass obstacles such as doorsteps or carpets easily. The most significant disadvantage of the platform is its lack of odometry sensors. Therefore, the odometry measurements were performed in a crude manner, by estimating the travelled distance and rotation angle by multiplying the elapsed time by the linear and rotation speed, respectively.

The platform was equipped with a Scanse Sweep LiDAR, which is a discontinued 360-degree range sensor. During the study, the LiDAR was set to collect scans with a 2 Hz rate.

The system tag was attached at the middle of the robot on a one-meter long wooden pole, which placed it at a height of 1.3 m. Such elevation is close to a tag worn on a lanyard and ensured that low furniture pieces such as bed frames, couches or tables would not negatively impact the RSS measurement results.

### 6.2. Environment Mapping

**Figure 9:**
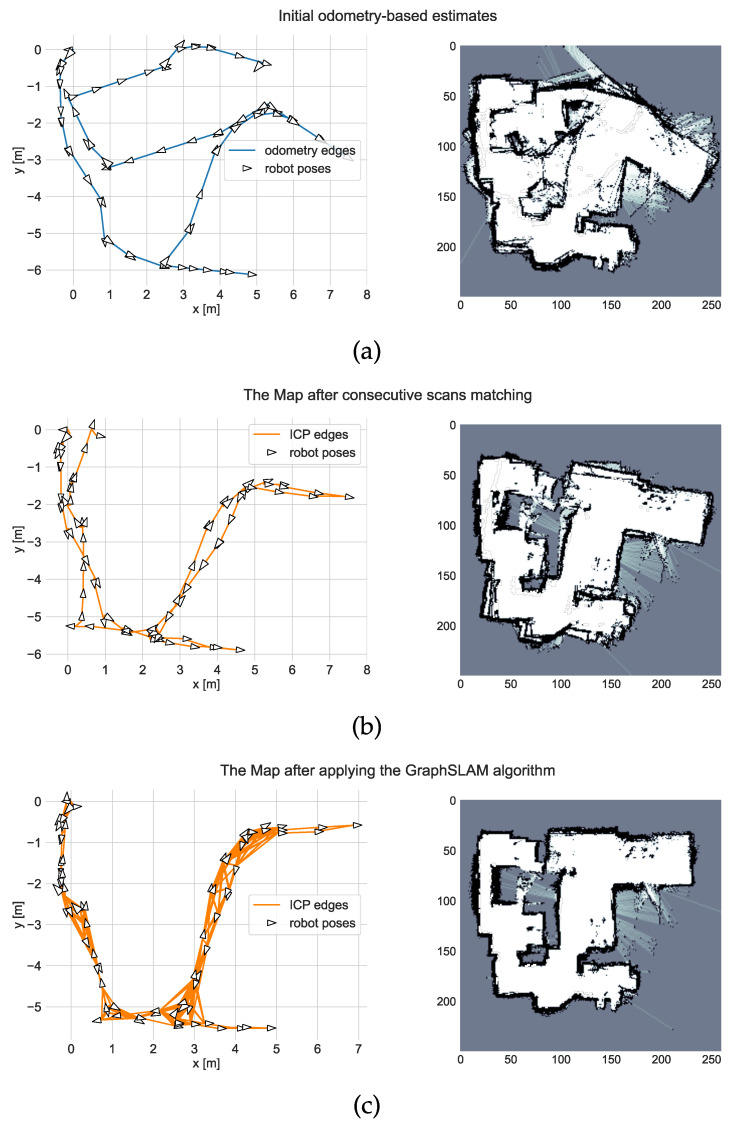
The results of the environment mapping: (**a**) Odometry-only estimate; (**b**) Estimate based on consecutive scans matching (78 ICP edges); (**c**) The final result of the GraphSLAM algorithm (349 ICP edges).

The first step of the experiments was environment mapping. The robot was driven through all of the rooms twice and performed 79 stationary scans. The results were processed using the proposed SLAM algorithm. The values of the tunable parameters used in the study are listed in Table 2. The mapping results and graph created at different steps of the algorithm are presented in Figure 9.

The line splitting threshold was set to 0.1 m to ensure that a line segment would not be split due to LiDAR sensor noise, which might be high in the case of close-range measurements. Filtering out the segments, which consisted of less than 15 points and were shorter than 0.2 m, discarded small segments, which might appear in the case of noisy parts of the scan.

The values of bearing and range tolerances allow the algorithm to detect line-correspondence even when the odometry estimate is not very accurate. The relatively low maximum distance between points taken for ICP fitting—0.1 m ensured that the algorithm will not match different, far away segments.

The requirements set for ICP candidates detection in the GraphSLAM part change throughout the algorithm. First, they were strict in choosing only scans, which were close to each other, and the initial guess on transformation would not be severely affected by the error propagation between the poses. The thresholds were changed later on to take into account the more distant scan pairs.

Due to the lack of typical sensors, such as wheel encoders, the odometry measurement results were inaccurate, and the initial estimate of the robot poses was poor. The map was better after considering the results of the consecutive scan matching but was still unfit for robot positioning. The final result of the GraphSLAM algorithm was satisfactory. The loops were correctly closed and the scans were aligned with much better accuracy.

The final map was manually transformed so that it overlaps with the construction plan of the apartment presented in Figure 7.

### 6.3. RSS Calibration

**Figure 10:**
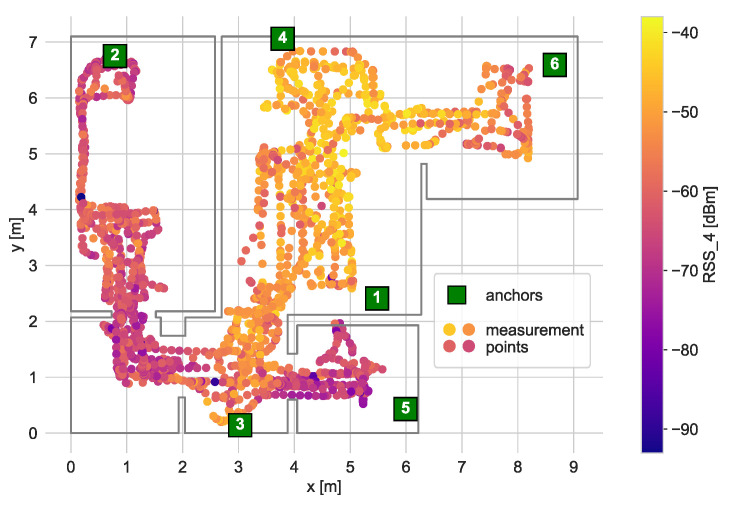
Locations of calibration measurements. The colours of the points reflect the measured RSS value by anchor 4.

The central part of the experiment was the system radio map calibration. This part of the experiment consisted of driving the robot across the apartment and registering the levels of BLE signals measured by the anchors. The calibration path and RSS levels measured for one of the anchors are presented in Figure 10.

The robot took measurements only in accessible places. Therefore, the number of measurements performed in the bedroom was smaller as a large bed frame takes a large portion of the area.

The registered RSS values were processed with all of the methods presented in Section 5. The methods were implemented in Python using NumPy, SciPy (for log-distance path loss model fitting), scikit-learn (for GPR and RF regression) and TensorFlow using Keras API (for Neural Network’s implementation).

**Figure 11:**
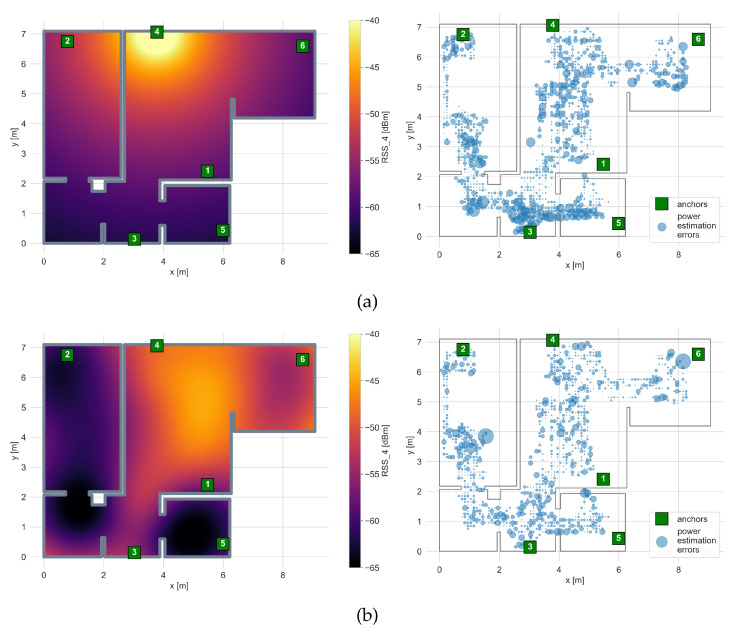
Radio maps for anchor 4 interpolated using (**a**) the log distance path loss model, (**b**) Gaussian Process regression. The size of the dots indicate the power estimation errors when compared to the calibration dataset.

The exemplary results of the radio map calibration for a log-distance path loss model and a Gaussian Process Regression are shown in Figure 11. The fitted γ values for the path loss models and the tuned hyperparameters of the GPR are presented in Table 3 and Table 4 respectively.

The fitted γ values are in the range of 2.3–3.11, which are similar values to those reported in the literature [37]. The typical log-distance path loss model is very simple as it does not consider additional power losses introduced by propagation through walls and obstacles. It is visible in the obtained radio map, where the difference between power levels on both sides of the living-room–bathroom wall is minimal, where in reality it is higher than 10 dB.

The values of the GPR model’s hyperparameters, for which the obtained results were the best, are shown in Table 4.

Thanks to the Gaussian Process’ ability to model more complex functions, the obtained radio map better reflects the signal distribution in the apartment. The power levels in distant areas separated by the multiple walls and obstacles are significantly lower, and thus the power estimation errors are smaller. However, it is still hard to model large, abrupt changes with the GPR model. As can be seen, the power estimation errors in the bottom area of the bedroom are still significant due to the area being shadowed by a large 65-inch TV screen located in the adjacent room.

In the case of the machine learning methods, the localisation was performed directly using the trained models, and the radio map creation is unnecessary. The hyperparameters of both algorithms were optimised. The topology of the implemented Neural Network and training parameters are presented in Table 5. The parameters of the used Random Forest regressor are shown in Table 6.

The Neural Network was implemented using Tensorflow with Keras API. The network consisted of an input layer with six inputs corresponding to the particular anchors, five hidden layers and an output layer. The most accurate model was achieved for batch size of 128 and the mean squared error loss function.

The Random Forest regressor was implemented using scikit-learn (RandomForestRegressor from ensemble library). The used model consisted of 100 trees. The nodes were split if there were more than 40 samples available and maximum of two randomly chosen features were used as a splitting candidate.

### 6.4. Positioning Results

**Figure 12:**
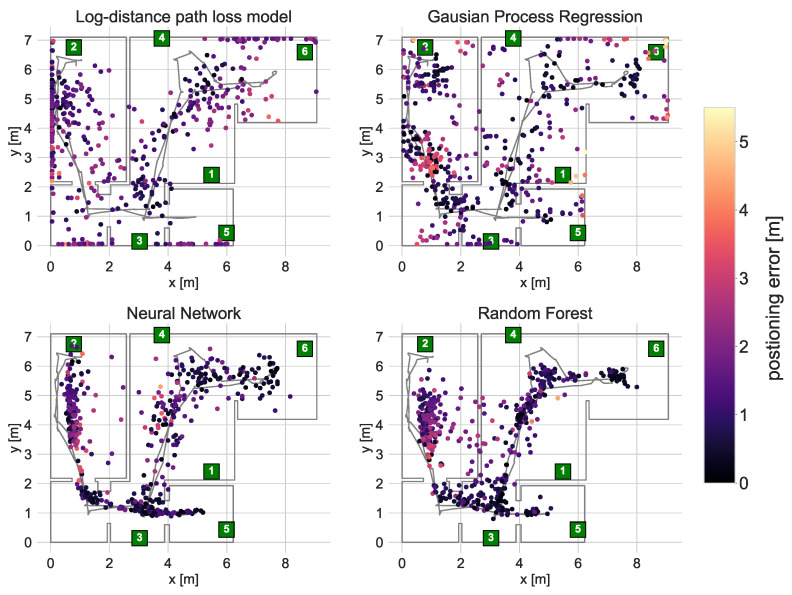
Locations of a moving robot derived using the calibrated models.

**Figure 13:**
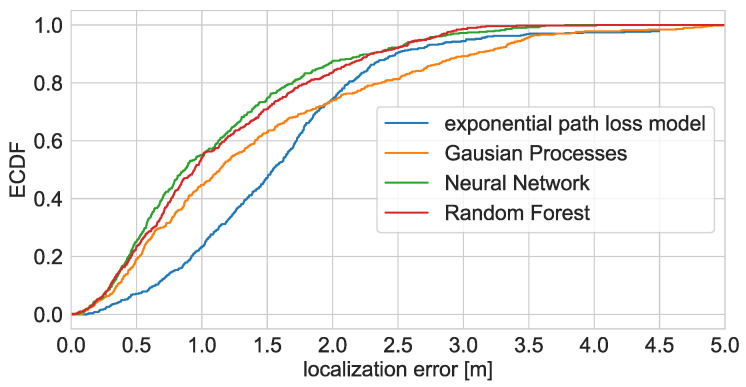
Empirical Cumulative Distribution Function of moving robot localisation errors.

The accuracy of the calibrated radio maps and trained regression models was tested by localising a moving robot and a person walking along a reference path. The robot localisation results are presented in Figure 12. The localisation error statistics and its Empirical Cumulative Distribution Functions are presented in Table 7 and Figure 13.

The most accurate robot localisation results were achieved using the Neural Network (ANN) and Random Forest Regression (RFR). The median error in both cases was below one meter, and the RMSE was less than 1.5 m. In both cases, it was possible to determine the trajectory of the robot. In the case of the RFR, the robot was not properly localised in the top area of the bedroom. This might result from the fact that the number of samples gathered there was relatively modest due to the area being small and cluttered with furniture, restricting robot movement. Given that, during the optimisation, the most favourable minimum split size was set to 40, the leaves of the trees might also include samples from the below area. The ANN allowed for proper localisation in all areas. However, the variance of the results is higher than in the case of the RFR.

The localisation results obtained using maps interpolated with GPR and log distance path loss model are much worse. In the case of the path loss model, many of the results are located at the outer apartment walls, where the estimated levels are the lowest. It is caused by the model’s simplicity and not taking wall attenuation into account.

The GPR obtained map yields better results. The results are not pulled towards the ends of the apartment, and it is possible to determine the room/area where the robot was located. However, the accuracy is still rather low as the resulting functions approximating the power distribution in the apartment resulting from the GPR are smooth and do not model abrupt changes in RSS well [38]. It is an important issue in the test environment due to multiple highly attenuating obstacles such as an elevator shaft or a large TV.

**Figure 14:**
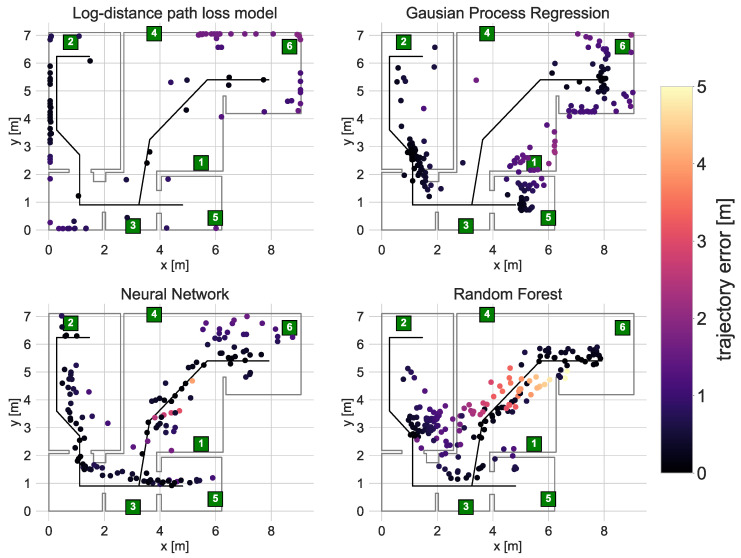
Locations of a walking person derived using the calibrated models.

**Figure 15:**
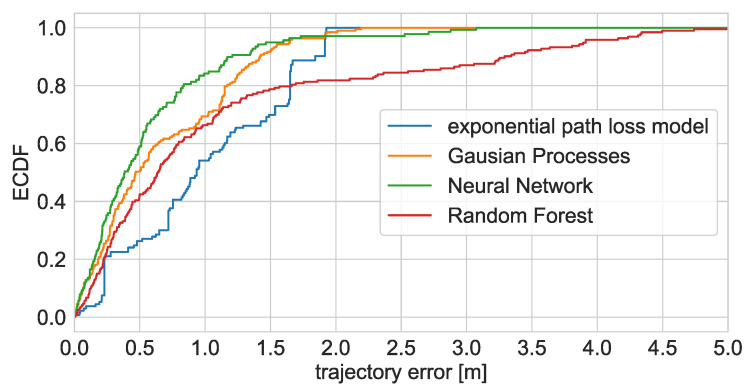
Empirical Cumulative Distribution Function of walking person localisation trajectory errors.

The second test of the proposed method consisted of localising a person walking through the apartment. The exemplary localisation results are presented in Figure 14. Because it was not possible to determine the exact person location at a given time, the localisation accuracy was evaluated based on the trajectory error defined as the smallest distance between the positioning result and the reference trajectory lines. The trajectory error ECDF and statistics are presented in Figure 15 and Table 8.

The person localisation results are less accurate than in the case of the robot. This was expected, as the created radio maps and trained models are based on the data gathered with the robot and those data do not take into account human body shadowing, which introduces several dB attenuations.

The best results were definitely achieved using the Neural Network. The obtained median trajectory error equalled 40 cm, and it was possible to reconstruct the walking trajectory properly. The quality of the results of the RFR, which in the case of robot localisation was on par with the ANN, is visibly worse for person localisation. The trained RFR model does not allow proper localisation in the wardrobe and the top area of the bedroom. Some of the introduced errors are as high as five meters. As with the robot localisation, the lower accuracy may be caused by a small number of calibration signatures gathered in those areas.

The solutions based on the interpolated radio maps did not work correctly in the person localisation scenario. As discussed in Section 6.3, the resulting radio maps do not adequately model wall attenuation (LDPL) and abrupt changes(LDPL and GPR). Adding the additional attenuation caused by the body shadowing, both models are not enough to localise the user appropriately.

In the case of the Log Distance Path Loss model, it was not possible to correctly derive user locations. As in Figure 12, the results tend to be pulled to the outer walls, where the predicted power levels are the lowest. This effect is additionally magnified by the body shadowing.

In the case of the GPR, the person is appropriately localised only in selected areas. This leads to misleading trajectory error statistics.

### 6.5. Comparison with Other Methods

The accuracy of the proposed method is at a similar level to those presented in the literature (Table 1). Compared to the method with targeted map updates, the accuracy is lower (1.1 m mean error for ANN compared to 0.6 m). The difference is understandable as the problematic regions (such as the top of the bedroom) were not additionally sampled after the calibration process. The median error is significantly better than in the case of the typical kNN fingerprinting presented in [33] (0.87 m compared to 1.22 m).

When it comes to localisation of a moving person, the median trajectory error is similar to that reported in [22] (0.4 vs. 0.42 m). It is worth noting that, in the referenced work, where the signatures were gathered with a hybrid BLE/UWB system and the resulting radio maps were personalised, the maps were interpolated using the GPR. In the case of the robot collection, the GPR map was barely usable (Figure 14. This proves that, in the case of person localisation, the body shadowing has a significant impact on the measured RSS values and it would be preferable if, for such applications, the data were gathered by the target users.

The robot solutions described in the literature achieve a slightly better accuracy. The worse performance might result from different environments where the measurements were taken and systems used. In [23] (median error 0.72 m), the tests were performed in a single room and an office space—the CDFs include results from both locations. In [24], the experiment was conducted in a single room.

## 7. Conclusions

This paper presents a complete radio map calibration method intended for use in RSS-based indoor positioning systems. The method covers all steps from environment mapping to radio map calibration based on the data gathered by a mobile robot. In the study, system calibration algorithms were tested: interpolating radio maps with a fitted log-distance path loss model and Gaussian Process Regression, Neural Network and Random Forest Regression.

The tests were performed with a BLE-based localisation system in a demanding propagation environment of a fully furnished apartment and have shown that the proposed method achieves a similar accuracy to that of the methods described in the literature. The median localisation error of a robot was about 0.87 m.

The proposed method can be treated as a possible alternative to other radio map calibration solutions described in the literature. Although it was tested with a BLE system, it can find multiple applications in systems based on different technologies. It may be especially helpful in places, where obtaining a big crowdsourced set is impossible due to a small number of users or if the calibration must be performed in a short amount of time.

## Figures and Tables

**Table 1:** Comparison of the reported localisation accuracy of selected methods.

Method	Median Error [m] ${}^{1}$	Method Features
**Bluetooth Fingerprinting**		
[23]	0.72	kNN, robot collected data at two test sites
[17]	1.1	DR with turn landmarks, radio map update
[12]	1.34	CNN for region estimation, magnetometer-based refinement
[13]	0.6 ${}^{2}$	targeted updates of low accuracy regions
[33]	1.22	typical kNN fingerprinting
[31]	in 2–3 m range	Neural Network and SVM regression
[22]	0.42–0.52 ${}^{3}$	UWB-based fingerprint locations; GPR; personalised maps
[21]	0.72-0.85 ${}^{2}$	UWB-based fingerprint locations; kNN, Random Forest
**WiFi Fingerprinting**		
[16]	2.90–3.00	DR with map pulling; fingerprints averaging
[9]	2.30–2.45	DR GraphSLAM, GP
[19]	0.93	GPR; MPEGP radio map update
[10]	1.40	GPR based on robot collected data
[20]	1.86	unlabelled dataset processed with MDS
[27]	2.1	GBDT-based altered AP detection and correction
[6]	1.42	DR with turn landmarks; fingerprints averaging
[24]	0.60	ASMF algorithm processing robot collected data
[26]	1.3	DR+MDS; unprocessed fingerprints
[18]	4.3	GraphSLAM with Virtual Wi-Fi landmarks
[25]	2.50	unprocessed robot-collected fingerprints for a few points

${}^{1}$ if not noted otherwise, ${}^{2}$ mean error, ${}^{3}$ trajectory error.

**Table 2:** SLAM algorithm parameters.

Parameter	Name	Value
line split threshold	$\delta_{th}$	0.1 m
minimum line length	$l_{min}$	0.2 m
minimum line points	$n_{min}$	15
bearing tolerance	$\Delta \phi_{th}$	0.3 rad
range tolerance	$\Delta r_{th}$	0.2 m
ICP points maximum distance	$\Delta x_{th}$	0.1 m
ICP pose maximum distance	$\Delta x_{th}$	1 m ${}^{1}$(2 m ${}^{2}$)
ICP minimum common gridmap	$c_{G}$	0.7 ${}^{1}$(0.6 ${}^{2}$)

${}^{1}$ For eight first GraphSLAM iterations, ${}^{2}$ From the eighth GraphSLAM iteration.

**Table 3:** The fitted path loss exponent γ parameter values.

Anchor	1	2	3	4	5	6
path loss exponent γ	3.11	3.07	2.61	2.39	3.07	2.31

**Table 4:** The tuned values of the GPR model parameters.

Hyper Parameter	Value
length-scale *l*	0.1
smoothness ν	1.5
noise level $\sigma^{2}$	0.2
constant value *c*	0.1

**Table 5:** Neural network topology and training parameters.

Network Topology	
input dimension	6
output dimension	2
hidden layers	5
no. of hidden layers neurons	64
input layer activation	ReLu
hidden layer activation	ReLu
output layer activation	linear
**Training Parameters**	
batch size	128
epochs	500
loss function	mean squared error
optimiser	adam

**Table 6:** The tuned values of the RF model parameters.

Hyper Parameter	Value
no. of trees	100
max features	2
min samples split	40

**Table 7:** The robot localisation error statistics [in metres].

Model	Mean	Q1	Median	Q3	Max	RMSE
Log-Distance Path Loss	1.54	1.02	1.53	1.97	4.5	1.7
Gaussian Process Regression	1.45	0.58	1.14	2.06	5.71	1.81
Neural Network	1.10	0.50	0.87	1.52	4.45	1.36
Random Forest	1.15	0.53	0.94	1.62	4.5	1.39

**Table 8:** The walking person trajectory error statistics [in meters].

Model	Mean	Q1	Median	Q3	Max	RMSE
Log-Distance Path Loss	1.00	0.48	0.93	1.65	1.93	1.17
Gaussian Process Regression	0.67	0.23	0.48	1.13	2.19	0.87
Neural Network	0.58	0.19	0.40	0.77	4.01	0.85
Random Forest	1.12	0.28	0.64	1.27	5.34	1.68

## Data Availability

The data used for the study can be found at https://zenodo.org/record/5457591 (accessed on 19 July 2021).

## Abbreviations

The following abbreviations are used in this manuscript:

ANN	Artificial Neural Network
ASMF	adaptive signal model fingerprinting
BLE	Bluetooth Low Energy
CDF	Cumulative Distribution Function
GP	Gaussian Process
GPR	Gaussian Process Regression
ICP	Iterative Closest Point
LiDAR	Light Detection and Ranging
LS	Least Squares
MPEGP	marginalized particle extended Gaussian Process
RFR	Random Forest Regression
RSS	Received Signal Strength
SVM	Support Vector Machine
SLAM	Simultaneous Location and Mapping
TWR	Two-Way Ranging
UKF	Unscented Kalman Filter
UWB	ultra-wideband
